# Susceptibility Genes and HLA for Cold Medicine-Related SJS/TEN with SOC

**DOI:** 10.3389/fgene.2022.912478

**Published:** 2022-07-11

**Authors:** Mayumi Ueta

**Affiliations:** Department of Ophthalmology, Kyoto Prefectural University of Medicine, Kyoto, Japan

**Keywords:** HLA, cold medicine, Stevens–Johnson syndrome, Toxic epidermal necrolysis, severe ocular complications, TLR3, PTGER3, IKZF1

## Abstract

We investigated the genetic predisposition for the pathogenesis of Stevens–Johnson syndrome/epidermal necrolysis with severe ocular complications (SJS/TEN with SOC). Cold medicines (CMs) including multi-ingredient cold-medications and non-steroidal anti-inflammatory drugs (NSAIDs) were implicated in the development of SJS/TEN with SOC. Studies on the association between HLA genotypes and CM-related SJS/TEN with SOC (CM-SJS/TEN with SOC) revealed an association with HLA-A*02:06 in the Japanese; it may be a marker in Koreans. HLA-B*44:03 was associated with the Japanese, Thais, and Indians; in Brazilians of European ancestry, it may be a positive marker. *PTGER3* is a susceptibility gene; HLA-A*02:06 and *PTGER3* polymorphisms exerted additive effects in Japanese and Korean patients. A genome-wide association study showed that *IKZF1* was associated with the Japanese. A meta-analysis including Japanese, Koreans, Indians, and Brazilians also revealed an association between CM-SJS/TEN with SOC and *IKZF1.* The upregulation of hsa-miR-628-3p in the plasma of SJS/TEN with SOC patients may suppress the expression of *TLR3* and innate immune-related genes. Not only CMs but also the interaction of *TLR3*, *PTGER3*, *IKZF1*, and *HLA* and maybe some microbial infections are necessary for the onset of SJS/TEN with SOC.

## Severe Ocular Complications of SJS/TEN

Stevens–Johnson syndrome (SJS) and its severe phenotype, toxic epidermal necrolysis (TEN), are acute inflammatory vesiculobullous reactions of the skin, mucosa of the ocular surface, oral cavity, and genitals. About half of SJS/TEN patients in the acute stage, who were diagnosed in burn units and/or by dermatologists, have had severe ocular complications (SOC) such as severe conjunctivitis with both ocular surface epithelial defects and pseudomembrane (Sotozono et al., 2015).

Burn unit physicians and dermatologists usually see SJS/TEN patients only in the acute stage since the skin lesions have healed after the acute stage (Yamane et al., 2007). On the other hand, since some SJS/TEN patients present with ocular sequelae such as severe dry eye and corneal opacity with vision disturbance (Sotozono et al., 2007), ophthalmologists tend to see these patients not only in the acute but also in the chronic stage.

In the acute stage, the ocular surface of SJS/TEN with SOC patients manifests severe conjunctivitis with both epithelial defects and pseudomembrane (Sotozono et al., 2009). In the chronic stage, many SJS/TEN with SOC patients suffer serious ocular sequelae such as vision disturbance due to severe dry eye and conjunctival invasion into the cornea (Sotozono et al., 2007).

SJS and TEN with SOC tend to be reported as “SJS” in ophthalmology (Ueta and Kinoshita, 2012), because it can be difficult for ophthalmologists to make a differential diagnosis of SJS or TEN in the chronic stage since the vesiculobullous skin lesions observed in the acute stage have healed, and ophthalmologists tend to diagnose SJS/TEN in their chronic stage based on a confirmed history of acute-onset high fever, serious mucocutaneous illness with skin eruptions, and the involvement of at least two mucosal sites including the ocular surface (Ueta et al., 2007a; Ueta et al., 2007b; Ueta et al., 2010a; Ueta et al., 2015a; Ueta et al., 2017a).

For more than 15 years, we focused on the genetic predisposition for and the pathogenesis of SJS/TEN with SOC. We found that cold medicines (CMs) including multi-ingredient cold medications and non-steroidal anti-inflammatory drugs (NSAIDs) such as acetaminophen and dipyrone are major causative drugs for SJS/TEN with SOC (Ueta et al., 2010a; Lee et al., 2017; Wakamatsu et al., 2017; Jongkhajornpong et al., 2018; Jongkhajornpong et al., 2020; Ma et al., 2021; Wakamatsu et al., 2021), although dermatologists and others reported that allopurinol (a uric acid-lowering drug) (Hung et al., 2005; Tohkin et al., 2013) and anticonvulsants such as carbamazepine (Chung et al., 2004; Kaniwa et al., 2010; McCormack et al., 2011; Mockenhaupt et al., 2019) are the main SJS/TEN-inciting drugs.

We have reported that about 80% of SJS/TEN with SOC patients seen at the Kyoto Prefectural University of Medicine developed SJS/TEN within several days after taking cold medicines (CMs) (Ueta et al., 2010a; Ueta et al., 2014a; Ueta et al., 2015a). Our Brazilian collaborators also found that 53% of their SJS/TEN with SOC patients had taken cold medicines (Wakamatsu et al., 2017) as had 69% of Thai (Jongkhajornpong et al., 2018) and 50% of Taiwanese patients (Ma et al., 2021). Our Korean collaborators suspected that NSAIDs and CMs were associated with SOC in their SJS/TEN patients (Lee et al., 2017). Thus, in patients of different ethnicities, ophthalmologists reported that CMs appear to be major causative drugs for SJS/TEN with SOC.

Because for SJS/TEN with SOC, the purpose of taking cold medicines including multi-ingredient cold medications and NSAIDs before their onset might be to combat the common cold, we also suspect that the onset of CM-related SJS/TEN with SOC was associated not only with cold medicines but also with putative microbial infection (Ueta and Kinoshita, 2012; Ueta, 2018; Ueta, 2021a).

Moreover, the associated HLA types vary among different causative drugs; for example, HLA-B* 58:01 for allopurinol (Hung et al., 2005; Tohkin et al., 2013), HLA-B*15:02 (Chung et al., 2004; Kaniwa et al., 2008; Kaniwa et al., 2013), HLA-A*31:01 (McCormack et al., 2011; Mockenhaupt et al., 2019), HLA-B*57:01 (Mockenhaupt et al., 2019) for carbamazepine, and HLA-A*02:06 and HLA-B*44:03 for CM-related SJS/TEN with SOC (Ueta et al., 2014a; Ueta et al., 2014b). Therefore, it is likely that pathogenesis is different among different causative drugs or between SJS/TEN with and without SOC (Ueta et al., 2014a). Here, we focus on CM-related SJS/TEN with SOC.

## HLA Association with CM-Related SJS/TEN with SOC

We have analyzed the association between CM-related SJS/TEN with SOC and HLA genotypes, and found that CM-related SJS/TEN with SOC was significantly associated with HLA-A*02:06 [151 patients, 639 normal controls; odds ratio (OR) = 5.6, *p* = 2.7 × 10^–20^] and with HLA-B*44:03 in the Japanese (151 patients, 639 normal controls; OR = 2.0, *p* = 1.3 × 10^–3^) (Ueta et al., 2014a). HLA-A*02:06 and HLA-B*44:03 were not associated with CM-related SJS/TEN without SOC, suggesting that a different *HLA* genotype plays a role in the development of SJS/TEN with and without SOC (Ueta et al., 2014a). Moreover, these *HLA* genotypes are not associated with CM-unrelated, that is, other medicine-related SJS/TEN with SOC (Ueta et al., 2014a).

We have suspected that the pathogenesis of SJS/TEN with SOC is different from the pathogenesis of SJS/TEN without SOC (Ueta and Kinoshita, 2012), since major causative drugs for SJS/TEN with SOC were different from those for SJS/TEN without SOC, and the HLA association with SJS/TEN with SOC was different from SJS/TEN without SOC.

For further investigation of the genetic predisposition for SJS/TEN with SOC, we have engaged in an international collaboration that included participants from Korea, Brazil, Thailand, Taiwan, India, and Japan.

Our Korean collaborators identified HLA-A*02:06 (40 patients, 120 controls; OR = 3.0, *p* = 0.0083) as potential positive markers for CM-related SJS/TEN with SOC in Korea as same as in Japan. They also reported that HLA-C*03:04 (40 patients, 120 controls; OR = 3.5, *p* = 0.010) might be a potential positive marker for CM-related SJS/TEN with SOC, and HLA-C*03:03 (40 patients, 120 controls; OR = 0.10, *p* = 0.0056) might be a possible indicator of the protection against CM-related SJS/TEN with SOC in Korea (Jun et al., 2019).

Our Brazilian collaborators reported HLA-A*66:01 as a potential marker for CM-related SJS/TEN with SOC in Brazilians (39 patients, 133 controls; OR = 24.0, *p* < 0.001) of both Pardo (19 patients, 66 controls; OR = 12.2, *p* = 0.03) and European ancestry (16 patients, 61 controls; OR = 21.2, *p* = 0.04), HLA-B*44:03 (16 patients, 61 controls; OR = 5.50, *p* = 0.01) and HLA-C*12:03 (16 patients, 61 controls; OR = 8.79, *p* = 0.008) may be markers only in individuals of European ancestry, and HLA-A*11:01 (39 patients, 133 controls; OR = 0.074, *p* = 0.008) may be a marker of resistance to CM-related SJS/TEN with SOC in the Brazilian population (Wakamatsu et al., 2017).

Our Thai collaborators reported that HLA-B*44:03 (49 patients, 159 controls; OR = 7.2, *p* < 0.0001) and HLA-C*07:01 (49 patients, 159 controls; OR = 6.1, *p* < 0.0001) were significantly associated with Thai CM-related SJS/TEN with SOC, and identified that the HLA-B*44:03 - HLA-C*07:01 haplotypes were a potential risk factor for CM-related SJS/TEN with SOC in their population (Jongkhajornpong et al., 2018).

Our Taiwanese collaborators reported that HLA-A*02:07 (13 patients, 98 controls; OR = 5.6, *p* = 0.016) was associated with Han Chinese CM-related SJS/TEN with SOC patients (Ma et al., 2021). As HLA-A*02:06 and HLA-A*02:07 are very similar peptides—they differ in only a single amino acid residue substitution—it is possible that the expression of HLA-A*02:07 but not of HLA-A*02:06 was associated with CM-related SJS/TEN with SOC in the Han Chinese population (Ma et al., 2021).

Our Indian collaborators reported that it was difficult to obtain a detailed history of disease onset from their SJS/TEN with SOC patients and in many patients; they could not identify causative drugs. However, an HLA analysis showed that HLA-A*33:03 (80 patients, 50 controls; OR = 3.4, *p* = 2.7 × 10^–3^), HLA-B*44:03 (80 patients, 50 controls; OR = 12.2, *p* = 7.3 × 10^–9^), and HLA-C*07:01 (80 patients, 50 controls; OR = 6.5, *p* = 4.4 × 10^–6^) were risk alleles, and haplotypes comprising HLA-B*44:03 and HLA-C*07:01 were strongly associated with SJS/TEN with SOC in the Indian population (80 patients, 50 controls; OR = 11.0, *p* = 1.1 × 10^–7^) (Kannabiran et al., 2017). We also reported that HLA-B*44:03 was strongly associated with CM-related SJS/TEN with SOC in the Indian population (20 patients, 55 controls; OR = 12.3, *p* = 1.1 × 10^–5^) (Ueta et al., 2014b).

**FIGURE 1:**
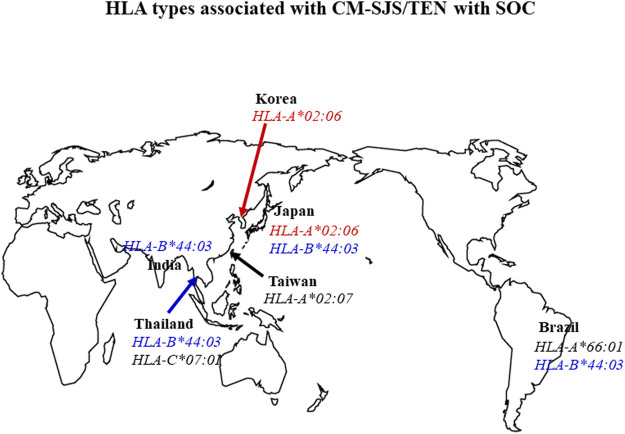
Summary of the association between HLA types and CM-related SJS/TEN with SOC. HLA-B*44:03 was significantly associated with CM-related SJS/TEN with SOC in Japanese, Brazilian, Indian, and Thai populations. HLA-A*02:06 was significantly associated with CM-related SJS/TEN with SOC in Japanese and Korean patients (OR: odds ratio OR).

In summary, HLA-B*44:03 was significantly associated with CM-related SJS/TEN with SOC in the Japanese (Ueta et al., 2014a), Brazilians (particularly in Caucasian Brazilians) (Ueta et al., 2014b; Wakamatsu et al., 2017), Indian patients (Ueta et al., 2014b; Kannabiran et al., 2017), and *Thais* (Jongkhajornpong et al., 2018; Jongkhajornpong et al., 2020). HLA-A*02:06 was significantly associated with CM-related SJS/TEN with SOC in the Japanese (Ueta et al., 2014a) and Koreans (Ueta et al., 2014b; Jun et al., 2019) (Figure 1).

CMs which combat the common cold include multi-ingredient CMs and NSAIDs such as ibuprofen and acetaminophen. We found that acetaminophen, present in various CMs, was the most frequently implicated causative drug in Japan (Ueta et al., 2014a; Ueta et al., 2019) and that HLA-A*02:06 was also strongly associated with acetaminophen-related SJS/TEN with SOC (80 patients, 113 controls; OR = 5.4, *p* = 8.0 × 10^–7^) (Ueta et al., 2019).

Similar to the United States and the United Kingdom, CMs, particularly acetaminophen (paracetamol), are widely used over-the-counter drugs in Thailand. Therefore, our Thai collaborators also investigated the HLA types in Thai patients with acetaminophen-related SJS/TEN with SOC. They found a significant association with HLA-A*33:03 (20 patients, 60 controls; OR = 5.4, *p* = 0.0030), HLA-B*44:03 (20 patients, 60 controls; OR = 9.0, *p* = 0.0004), HLA-C*07:01 (20 patients, 60 controls; OR = 9.3, *p* = 0.0002), and the HLA-B*44:03–HLA-C*07:01 haplotype (20 patients, 60 controls; OR = 9.0, *p* < 0.001) (Jongkhajornpong et al., 2020). This suggests that these HLA types play a role in the pathogenesis of SOC in acetaminophen-related SJS/TEN.

Our Brazilian collaborators found that among CMs, dipyrone, classified as an anti-inflammatory drug and widely used, was the main drug responsible for inciting SJS/TEN with SOC. They identified HLA-B*44:03 and HLA-DQB1*04:02 as potential risk factors for dipyrone-related SJS/TEN with SOC in the Brazilian population of European ancestry, and HLA-C*05:01 as a potential risk factor for dipyrone-related SJS/TEN with SOC in the Pardo Brazilian population (Wakamatsu et al., 2021).

We have suspected that a common function of CMs such as acetaminophen and dipyrone is highly implicated in the onset of SJS/TEN with SOC (Ueta et al., 2010a; Ueta, 2016; Ueta, 2018; Ueta, 2020; Ueta, 2021a; Ueta, 2021b).

## Susceptibility Genes for CM-Related SJS/TEN With *SOC*

### EP3 (PTGER3 Gene)

The common function of CMs is the suppression of prostaglandin E_2_ (PGE_2_) production. We have suggested that the common function of CMs might be important for the onset of CM-related SJS/TEN with SOC (Ueta et al., 2010a; Ueta, 2016; Ueta, 2018; Ueta, 2020; Ueta, 2021a; Ueta, 2021b) because PGE_2_ suppresses mucocutaneous inflammation (Kunikata et al., 2005; Ueta et al., 2009a; Honda et al., 2009); PGE_2_ acts on EP3 (PGE_2_ receptor 3) in the epidermis (Honda et al., 2009) and the mucosal epithelium such as the conjunctival (Ueta et al., 2009a) and tracheal epithelium (Kunikata et al., 2005), and it negatively regulates mucocutaneous inflammation (Kunikata et al., 2005; Ueta et al., 2009a; Honda et al., 2009). We have suspected that CMs including acetaminophen and NSAIDs could upregulate inflammatory responses by suppressing the production of PGE_2_ which suppresses mucocutaneous inflammation, that they augment abnormal immune responses, and that they elicit the induction of SJS/TEN with SOC (Ueta et al., 2010a; Ueta, 2016; Ueta, 2018; Ueta, 2020; Ueta, 2021a; Ueta, 2021b).

*PTGER3* is the gene of EP3. We also found that *PTGER3* is a susceptibility gene for CM-related SJS/TEN with SOC (Ueta et al., 2010a) and that HLA-A*02:06 and *PTGER3* polymorphisms exerted additive effects in Japanese and Korean patients with CM-related SJS/TEN with SOC (OR = 10.8 and 14.2, respectively) (Ueta et al., 2015b).

Our investigation of EP3 protein expression on the human ocular surface showed that the EP3 protein level was much lower in the conjunctival epithelium of patients with SJS/TEN with SOC than in the controls, that is, patients with conjunctival chalasis or chemical burns (Ueta et al., 2010a; Ueta et al., 2011a). This suggests that EP3 expression might be strongly downregulated on the ocular surface of patients with SJS/TEN with SOC and that the downregulation of EP3 protein expression might contribute to ocular surface inflammation in these patients (Ueta et al., 2010a; Ueta et al., 2011a; Ueta and Kinoshita, 2012; Ueta, 2021a).

## IKAROS (*IKZF1* Gene)

We also have studied other susceptibility genes for CM-related SJS/TEN with SOC using a genome-wide association study (GWAS) with Affymetrix Axiom Genome-Wide ASI 1 Array. Our study with 117 Japanese patients with CM-related SJS/TEN with SOC and 691 controls showed that the *IKZF1* gene was strongly associated with CM-related SJS/TEN with SOC in Japanese individuals (Ueta et al., 2015a). We found that a meta-analysis using samples from Japanese, Korean, Indian, and Brazilian patients revealed a significant genome-wide association between CM-related SJS/TEN with SOC and *IKZF1* [rs4917014 (G vs. T), OR = 0.5, *p* = 8.5 × 10^–11^] (Ueta et al., 2015a). We also analyzed the association between *IKZF1* single-nucleotide polymorphisms (SNPs) and Thai patients with CM-related SJS/TEN with SOC, and found that the *IKZF1* SNP rs4917014 (G vs. T) was also significantly associated with Thai patients with CM-related SJS/TEN with SOC (Chantaren et al., 2019). These findings suggest *IKZF1* is a universal marker for susceptibility to CM-related SJS/TEN with SOC (Ueta et al., 2015a; Chantaren et al., 2019).

Because our functional analysis of *IKZF1* SNPs suggested the enhancement of the function of the *IKZF1* gene in CM-related SJS/TEN with SOC (Ueta et al., 2015a), we produced K5-*Ikzf1*-EGFP transgenic (*Ikzf1*Tg) mice by introducing the Ik1 isoform into their cells expressing keratin 5, which is expressed in the epithelial tissues of, for example, the epidermis and conjunctiva. We found that mucocutaneous inflammation was exacerbated in *Ikzf1*Tg mice (Ueta et al., 2018), in which keratinocyte and mucosal epithelium including conjunctiva strongly expressed IKAROS, the protein of the *IKZF1* gene. They developed not only dermatitis but also blepharoconjunctivitis. SJS/TEN with SOC in the acute stage shows not only skin and ocular surface inflammation but also oral mucosal erosion and paronychia. Our histology studies on *Ikzf1*Tg mice also showed not only dermatitis but also inflammation of their tongue tissue, blepharoconjunctiva, and paronychia, similar patients with SJS/TEN with SOC in the acute stage (Ueta et al., 2018). Thus, we concluded that *IKZF1* plays a critical role in maintaining mucocutaneous homeostasis (Ueta et al., 2018). The association between *IKZF1* SNPs and CM-related SJS/TEN with SOC suggests that *IKZF1* could strongly contribute to the pathogenesis of CM-related SJS/TEN with SOC (Ueta, 2018; Ueta, 2020; Ueta, 2021a).

## Abnormal Innate Immunity is Involved in Patients with SJS/TEN with SOC

Among TLR1–TLR10, TLR3 is expressed most strongly in the ocular surface epithelium such as conjunctiva and cornea, which is more intense than that in mononuclear cells (Ueta et al., 2005; Ueta and Kinoshita, 2010). TLR3 recognizes dsRNA, a component of the life-cycle of most viruses, and is a member of the toll-like receptor family that is important for innate immunity, and could induce pro-inflammatory cytokines and IFN-β on the ocular surface (Ueta et al., 2005; Ueta et al., 2010b; Ueta and Kinoshita, 2010). Using the candidate-gene approach, we analyzed TLR3 gene polymorphisms and found that several TLR3 SNPs were significantly associated with CM-related SJS/TEN with SOC (Ueta et al., 2007a; Ueta et al., 2012a; Ueta et al., 2012b). Our investigations of TLR3 gene functions using TLR3 transgenic (TLR3Tg) and TLR3 knock-out (KO) mice showed that the rate of ocular surface inflammation was significantly increased in TLR3Tg and significantly decreased in TLR3-KO mice (Ueta et al., 2009b), suggesting that TLR3 positively regulates ocular surface inflammation (Ueta et al., 2009b). TLR3 is expressed in the epidermis of the skin and it positively regulates skin inflammation (Nakamura et al., 2015; Yasuike et al., 2017), and it is possible that innate immunity such as TLR3 might contribute to mucocutaneous inflammation seen in SJS/TEN with SOC (Ueta and Kinoshita, 2012; Ueta, 2016; Ueta, 2018; Ueta, 2021a).

In patients with SJS/TEN with SOC, the plasma level of miR-628-3p miRNA was significantly elevated and this miRNA could silence the mRNA expressions of pathogen-associated molecular patterns (PAMPs) of TLR3, RIG-I, MDA5, and other innate immune-related molecules, such as IFI44L, CXCL11, TNFSF10, RSAD2, CXCL10, and CCL8 (Ueta et al., 2021). Consequently, the upregulation of hsa-miR-628-3p in the plasma of SJS/TEN with SOC patients may suppress *TLR3* gene expression and the expression of innate immune-related genes. On the other hand, we also found that in the conjunctival epithelium of SJS/TEN with SOC patients, hsa-miR-628-3p was downregulated, suggesting that its systemic (plasma) upregulation may compensate for its local (ocular surface) downregulation (Ueta et al., 2021). Because hsa-miR628p can regulate innate immunity, the upregulation of hsa-miR-628-3p in the plasma of SJS/TEN with SOC patients supports our hypothesis that abnormal innate immunity was observed in SJS/TEN patients with SOC.

The examination of tear cytokines of patients with SJS/TEN with SOC in the chronic stage showed that CXCL10 was significantly downregulated (Ueta et al., 2017b). As CXCL10 is highly induced by the *TLR3* ligand poly(I:C) in human corneal and conjunctival epithelial cells (Ueta et al., 2010b), it is possible that abnormal innate immunity is involved in the presence of *TLR3* on the ocular surface of SJS/TEN with SOC (Ueta and Kinoshita, 2012; Ueta, 2016; Ueta, 2018; Ueta, 2021a).

The susceptibility genes for CM-related SJS/TEN with SOC, *PTGER3,* and *IKZF1* also have functional interactions with TLR3; EP3 (*PTGER3)* negatively regulated *TLR3*-dependent ocular surface inflammation (Ueta et al., 2011b; Ueta et al., 2012a; Ueta et al., 2012c); and *IKZF1* mRNA was upregulated by *TLR3* in human epidermal keratinocytes and conjunctival epithelial cells (Ueta et al., 2018).

These combined findings suggest that abnormal innate immunity could strongly contribute to the etiology of SJS/TEN with SOC (Ueta and Kinoshita, 2012; Ueta, 2016; Ueta, 2018; Ueta, 2020; Ueta, 2021a).

Although there are some reports on oligoclonal T cell populations showing HLA restriction and drug reactivity for HLA-B*58:01 restricted allopurinol-SJS/TEN (Lin et al., 2015) and HLA-B*15:02 restricted carbamazepine-SJS/TEN (Wei et al., 2012), there are few reports on T cell-mediated mechanisms in CM-related SJS/TEN with SOC, suggesting that more investigations are required to elucidate the pathogenic mechanisms of CM-related SJS/TEN with SOC.

**FIGURE 2:**
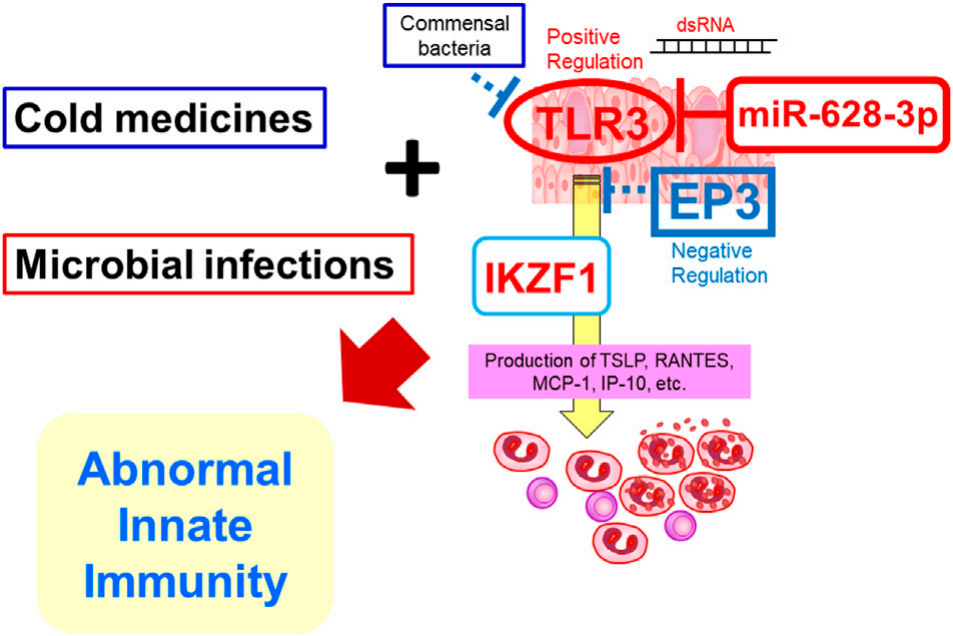
Abnormal innate immunity may be involved in the development of SJS/TEN with SOC.

Since CM-related SJS/TEN with SOC developed in patients after taking CMs for the common cold due to some viral or *mycoplasma* infection, we suspected that not only CMs, miRNAs such as hsa-miR-628-3p, and the interaction of susceptibility genes such as *TLR3, PTGER3* (which ligand, PGE_2_ is downregulated by cold medicines such as NSAIDs, acetaminophen, and dipyrone), and *IKZF1* but also some microbial infections are important and necessary for triggering the onset of SJS/TEN with SOC (Ueta and Kinoshita, 2012; Ueta, 2016; Ueta, 2018; Ueta, 2020; Ueta, 2021a) (Figure 2).

Despite the genetic diversity of CM-related SJS/TEN with SOC among different ethnic groups, we need to continue to identify the genetic predisposition for SJS/TEN with SOC to prevent its onset and to reduce the incidence of blindness due to SJS/TEN with SOC.

Since CM-related SJS/TEN with SOC is a rare condition with a complex genetic background, it is reasonable to posit the presence of multiplicative interactions of HLA and susceptibility genes such as HLA-A and *TLR3* (Ueta et al., 2012a), HLA-A and REC14 ^32^, and HLA-A and *PTGER3* (Ueta et al., 2015b), and it is possible that multiple susceptibility genes for CM-related SJS/TEN with SOC are involved in forming functional networks. An imbalance in these genes may trigger mucocutaneous inflammation seen in patients with CM-related SJS/TEN with SOC.

As our investigations identified HLA and several SNP sets with a high OR, their use may help alert the possibility of SJS/TEN with SOC onset.
